# A Case of Cowpox Presenting as Progressive Unilateral Facial Swelling and Fever Without Any Skin Lesions

**DOI:** 10.1155/crdi/4441632

**Published:** 2026-06-13

**Authors:** Oliver T. R. Toovey, Simon C. Wooding, Amy Belfield, Jenna Furneaux, Nicola Eisenhauer, Tommy Rampling

**Affiliations:** ^1^ Department of Virology, University Hospitals of Leicester NHS Trust, Leicester, UK, nhs.uk; ^2^ College of Life Sciences, University of Leicester, Leicester, UK, le.ac.uk; ^3^ Long Clawson Medical Practice, Melton Mowbray, UK; ^4^ Rare and Imported Pathogens Laboratory, UK Health Security Agency, Porton Down, Salisbury, UK; ^5^ University of Bristol Veterinary School, Bristol, UK; ^6^ Hospital for Tropical Diseases, University College London Hospitals NHS Foundation Trust, London, UK, nhs.uk

## Abstract

**Introduction:**

Although a rare infection, sporadic human cases of cowpox do occur in endemic areas, often through contact with an infected cat. Cats are also incidental hosts but provide an important intermediary to the animal reservoir for cowpox, which in the United Kingdom (UK) includes bank and field voles.

**Case Discussion:**

We present a case of cowpox in an immunocompetent 25‐year‐old male who developed a progressive unilateral facial swelling over the course of 10 days associated with pyrexia. He never developed any lesions, nor did he develop any necrotising lymphadenitis as has been described in previous such cases without visible lesions. He had been exposed to his cat at a time when the cat only displayed eye disease and had not been exposed during the phase of the cat’s illness when it had cutaneous manifestations. Both patient and cat made a full recovery.

**Conclusion:**

Cases of cowpox in immunocompetent patients typically involve distinctive skin lesions at the site of the inoculation, but facial swelling with or without lymphadenitis can occur.

## 1. Introduction

Cowpox is a member of the *orthopoxvirus* genus which also includes smallpox (variola), vaccinia and mpox virus (the cause of mpox). It is a large (250 × 200 nm) [1], enveloped double‐stranded DNA (dsDNA) virus. Poxviruses as causative agents of disease were an early discovery on account of their large size, allowing detection by light microscopy in 1886 by John Buist prior to the invention of electron microscopy. Confirmation of Enrique Paschen and Adelchi Negri’s hypothesis that these visualised viral particles were the cause of disease came with John Ledingham’s work in 1931 [2].

The disease cowpox has been known for centuries, and it was in the 1770s in England that it was first recorded that milkmaids who contracted cowpox and recovered were immune to further cowpox infections as well as smallpox. As a result, Dr Edward Jenner in 1796 famously inoculated the first patient with cowpox to provide immunity against the more deadly smallpox [2]. The vaccinia virus known today was once thought to be derived from cowpox, but genomic analysis has shown it to be more closely related to horsepox [3]. The cross‐protection of the vaccinia vaccine across the orthopox family was pivotal to the successful eradication of smallpox but also the global response to the recent mpox epidemics [4].

Generally, cowpox infection results in solitary skin lesions which occur at the point of inoculation through existing skin breaks. These have a characteristically haemorrhagic appearance which would typically trigger a clinical suspicion with supportive exposure history. Fever and lethargy are common systemic symptoms, and there can be vomiting and/or sore throat [5]. Cowpox is a self‐limiting infection, and supportive care is typically all that is required although systemic, even fatal, disease can and does occur in immunocompromised patients [5], while infection during pregnancy can lead to foetal loss [6].

Zoonotic orthopox viruses have seen a resurgence in recent decades, possibly related to the cessation of smallpox vaccination programmes since the successful smallpox eradication program. Increased contact between humans and the animal reservoirs of mpox through deforestation and habitat loss is also thought to have played a role in recent epidemics. Cowpox has remained a rare infection, however, with the principal animal reservoir, bank voles, short‐tailed field voles, and wood mice [7], being infrequently encountered by humans. Most human cases occur following infections in cats that serve as an intermediate host therefore. Suspected human‐to‐human transmission has been documented on at least one occasion in the literature [8]. We describe a case of cowpox in an immunocompetent male presenting without the typical lesions of this disease where the clue to the diagnosis came from the history of exposure to an unwell cat.

## 2. Case Presentation

A 25‐year‐old male, agricultural worker presented to his general practitioner (GP) with a 9‐day history of fever and right‐sided facial swelling. His symptoms started when he first noticed a lump under the right side of his jaw with some associated swelling in the same area. He became febrile with night sweats that evening and remained unwell the next day with ongoing fevers, feeling lightheaded, nauseous and generally fatigued. His symptoms persisted so he attended a local emergency department where he was given pyrexia management advice and discharged. By Day 5 of his illness, he felt that his fever was starting to improve but noticed progression of the swelling superiorly. By Day 8, the lower right half of his face was swollen such that he could not smile on that side. His right naris was swollen almost shut.

On Day 9 of the illness, he saw his GP, by which time his right inferior eye lid was swollen also. Despite feeling that his fever had improved, his temperature recorded in the practice was 38.5°C. On examination, he had mild swelling to the right side of his face with mild overlying erythema. The swelling was diffuse with no palpable masses. Examination of the right nostril was limited, but there was fluid visible as would be expected with congestion. There was no inflammation of the right eye on direct inspection. He did not have any lesions in the mouth or pharynx though his right tonsil was slightly enlarged but not inflamed. He also had no palpable lymphadenopathy.

He is usually fit and well and reported no foreign travel in the last 6 months. He had received all his routine childhood vaccinations. He had no new sexual partners in the preceding months. He has never smoked and consumes occasional alcohol. He had no unwell human contacts but had recently been attending the veterinarian with his cat. His cat, which is a free‐roaming domesticated cat, had become unwell 2 weeks prior to his disease onset, initially starting with bilateral eyelid swelling. The cat was prescribed antibiotic eye drops and eye bathes which the patient administered. While the patient was subsequently away for work, the cat required a return to the vet due to a failure to improve, and by Day 11 of the cat’s illness, it had developed pustular, umbilicated skin lesions. Swabs were taken by the vet, and a diagnosis of cowpox was confirmed by qPCR. The diagnosis was confirmed on day of the patient’s presentation to his GP, and as a result, the GP arranged testing in consultation with the local virologist who liaised with the Rare and Imported Pathogens Laboratory (RIPL), UK Health Security Agency. As the patient had not developed any skin lesions, a swab was taken from the naris on the affected side. A generic viral transport media swab was not immediately available to the GP, so an Aptima multitest swab was utilised and transported at room temperature. The differential diagnosis at this point also included a facial cellulitis/erysipelas, so a course of oral co‐amoxiclav 625 mg TDS for 7 days was prescribed. By the following week, his symptoms were much improved, and his fever had resolved completely. He never developed any lesions. Testing by RIPL confirmed that *orthopoxvirus* DNA was detected, probable cowpox. The cat also made a full recovery not long after the patient.

The patient’s only residual symptom at the time of his result was some right‐sided nasal congestion. The swelling had all resolved, and the patient had noted a small amount of dried blood in his right nostril but could not see any lesions. Given symptom resolution, he did not undergo nasendoscopy to exclude a lesion in the nasopharynx. The patient had not taken any photographs of either his own symptoms or those of his cats, and clinical photography was not available in the practice where he was seen.

## 3. Discussion

The patient never came into contact with the cat when it had lesions but had close contact at the start of the cat’s illness through the administration of eye drops and bathing the eyes. As previously hypothesised in the literature, route of inoculation likely determines disease presentation [9]. Contact with lesions typically results in lesions at the site of inoculation, but inhalation of virus during the early phase of the illness in the source mammal may have led to the patient’s atypical presentation. Unlike in the case published by Pahlitzsch et al. which involved a similar presentation of facial swelling, our patient did not, however, develop any necrotising lymphadenitis and recovered much more quickly [9]. Baxby et al. previously hypothesised the existence of these milder cases going undiagnosed as part of the spectrum of cowpox disease [5], but the true burden of mild/asymptomatic infection is difficult to quantify due to the lack of available serological assays with the ability to differentiate between specific *orthopoxviruses*.

### 3.1. Diagnostic Testing

Testing for *orthopoxviruses*, including Cowpox, in the United Kingdom is undertaken at the RIPL, UK Health Security Agency, Porton Down, United Kingdom. Only 3 cases of cowpox have been diagnosed between October 2023 and October 2024 via PCR testing at RIPL. The RIPL poxvirus testing algorithm comprises of PCRs to differentiate between the Poxviridae family, including a pan‐*orthopoxvirus*, *parapoxvirus* and monkeypox virus assays. The patient’s viral swab was inactivated using Roche MagNA Pure 96 external lysis buffer and extracted on the Roche MagNA Pure 96 platform. Extract was initially tested for Orthopox DNA PCR and monkeypox virus DNA PCR, using two assays based on published oligonucleotide sequences [10, 11] but optimised to conform with RIPL diagnostic mastermix and cycling conditions. The Orthopox DNA PCR was strongly positive with CT value 18.6; however, the mpox virus DNA PCR was negative. Two further PCR assays were performed to differentiate the orthopox‐positive PCR result to determine *orthopoxvirus* species, targeting the VETF and rpo18 gene, also based on a published assay [12]. Variola virus identification (Rpo18 PCR) was also negative. Orthopox identification (VETF PCR) had a melt curve of 50°C in keeping with a melt curve for monkeypox and a subset of cowpox (inc Brighton red strain), and due to the negative monkeypox virus DNA PCR, the result was reported as probable cowpox in the absence of an available specific cowpox virus DNA PCR at RIPL. Sequencing confirmation was not available.

### 3.2. Limitations

The improvement in the patient’s condition did coincide with the administration of the co‐amoxiclav, and we cannot, therefore, say for sure that the facial erythema and swelling were not due to a bacterial super‐infection which responded to the antibiotics. This, therefore, remains an important alternative explanation to our hypothesis that the improvement reflected the natural course of a self‐limiting viral infection. Other differentials beyond those already mentioned included glandular fever; however, with the prominent facial swelling and lack of lymphadenopathy, this differential was discarded, and a viral parotitis, which was felt unlikely given his up‐to‐date vaccination record and lack of palpable gland swelling.

We concede that the lack of clinical photography will make it difficult for clinicians to consider for themselves the validity of our conclusions in this case report. Moreover, due to the patient only being seen in primary care, more intensive investigation, such as nasendoscopy to exclude an occult mucosal lesion, could not be performed. Moreover, we could not justify arranging repeat testing to confirm the diagnosis with a cowpox‐specific DNA PCR given subsequent clinical improvement, and we, therefore, highlight here the further limitation that results, but note the high pretest probability given the significant exposure to confirmed cowpox without any risk exposure for other orthopox infection preceding the patient’s illness.

### 3.3. Monitoring Incidence

Cowpox is not a notifiable disease in the United Kingdom, either for human cases via the United Kingdom Health Security Agency (UKHSA) or for animal cases via the Animal and Plant Health Agency (APHA). The former agency does, however, utilise a Human Animal Infection Risk Surveillance (HAIRS) group assessment algorithm, with which cowpox would be classed as a Level 3 zoonotic infection. Level 3 involves pathogens that can cause human disease but for which there is no confirmed human‐to‐human spread, which would otherwise escalate it to Level 4 [13].

### 3.4. Prevention and Treatment

Given the main route of transmission of cowpox to humans is via cats [14], veterinary advice for owners regarding infection prevention measures is key. Indeed, recommendations are set out by the European Advisory Board on Cat Diseases on what measures owners should take to avoid contracting cowpox from their cat when the diagnosis is made [15]. In our case, the first veterinary contact was when the cat only had conjunctivitis, and so, there was, therefore, no clinical suspicion of cowpox at that time. No licensed vaccination or antivirals have been developed for cowpox. However, it is unknown whether smallpox vaccine would provide any cross‐protection as it has been shown to do for mpox disease [16]. Similarly, recent evidence has shown both tecovirimat and brincidofovir which were originally stockpiled in the United States for the treatment of smallpox have some efficacy against mpox disease [4]. It is plausible that such treatments could be considered in the case of severe cowpox infection on that basis, but there is no evidence available for this.

## 4. Conclusion

We have presented a rarely reported case of cowpox presenting without the characteristic skin lesion and following a generally mild clinical course. A high index of clinical suspicion for cowpox is required when assessing patients who have been in close contact with cats with the diagnosis, even in the absence of the characteristic skin lesions.

## Funding

No funding was received for this manuscript.

## Consent

Written informed consent for publication of this case report was obtained from the patient and is held by the corresponding author.

## Conflicts of Interest

The authors declare no conflicts of interest.

## Data Availability

The data that support the findings of this study are available on request from the corresponding author. The data are not publicly available due to privacy or ethical restrictions.
